# Transverse carcinoma after Miles operation: a case in which preoperative evaluation was assisted by computed tomographic colonography

**DOI:** 10.1186/s12957-016-0872-y

**Published:** 2016-04-19

**Authors:** Daisuke Ito, Masanori Teruya, Shojiro Hata, Kaoru Kobayashi, Michio Kaminishi

**Affiliations:** Department of Gastrointestinal Surgery, Showa General Hospital, 8-1-1, Hanakoganei, Kodaira, Tokyo, 187-8510 Japan

**Keywords:** Colon cancer, Colonography, Colostomy, Computed tomography

## Abstract

**Background:**

There were only few case reports in which CTC was performed in patients with colostomy.

**Case Presentation:**

A 68-year-old man was admitted with right abdominal pain and bloody stool that had been present for 2 weeks prior to admission. His medical history included abdominoperineal rectal resection with permanent sigmoid stoma (Miles operation). Colonoscopy showed a sub-occlusive tumor in the transverse colon but provided no information about the proximal colon. Thus, computed tomographic colonography (CTC) was planned to assist our examination of the proximal colon under sigmoid colostomy. CTC revealed the apple core sign in the hepatic flexure, without any evident tumor in the proximal colon. Therefore, we performed transverse colectomy and lymph node dissection, preserving a part of the ascending colon and Bauhin valve.

**Conclusion:**

CTC examination can be an effective means of preoperatively evaluating the proximal colon in patients with occlusive tumor. Further, CTC examination was technically feasible through a sigmoid stoma.

## Background

As first described by Vining in 1994 [1], computed tomographic colonography (CTC, also known as virtual colonoscopy or CT colonography) provides a computer-simulated endoluminal perspective on the air-filled distended colon. CTC can also be used to visualize the lumen of the colorectum in various three-dimensional views. Therefore, CTC can be helpful even though the bowel is narrowed or obstructed for any reason, such as by a large tumor [2, 3].

Despite these advantages, technical difficulty usually prevents CTC from being performed in patients with colostomy. Published reports have briefly described a few cases in which CTC was performed in patients with colostomy [4, 5].

In this report, we describe a case of transverse carcinoma after Miles operation, in which CTC provided effective assistance during preoperative evaluation.

## Case presentation

**Fig. 1:**
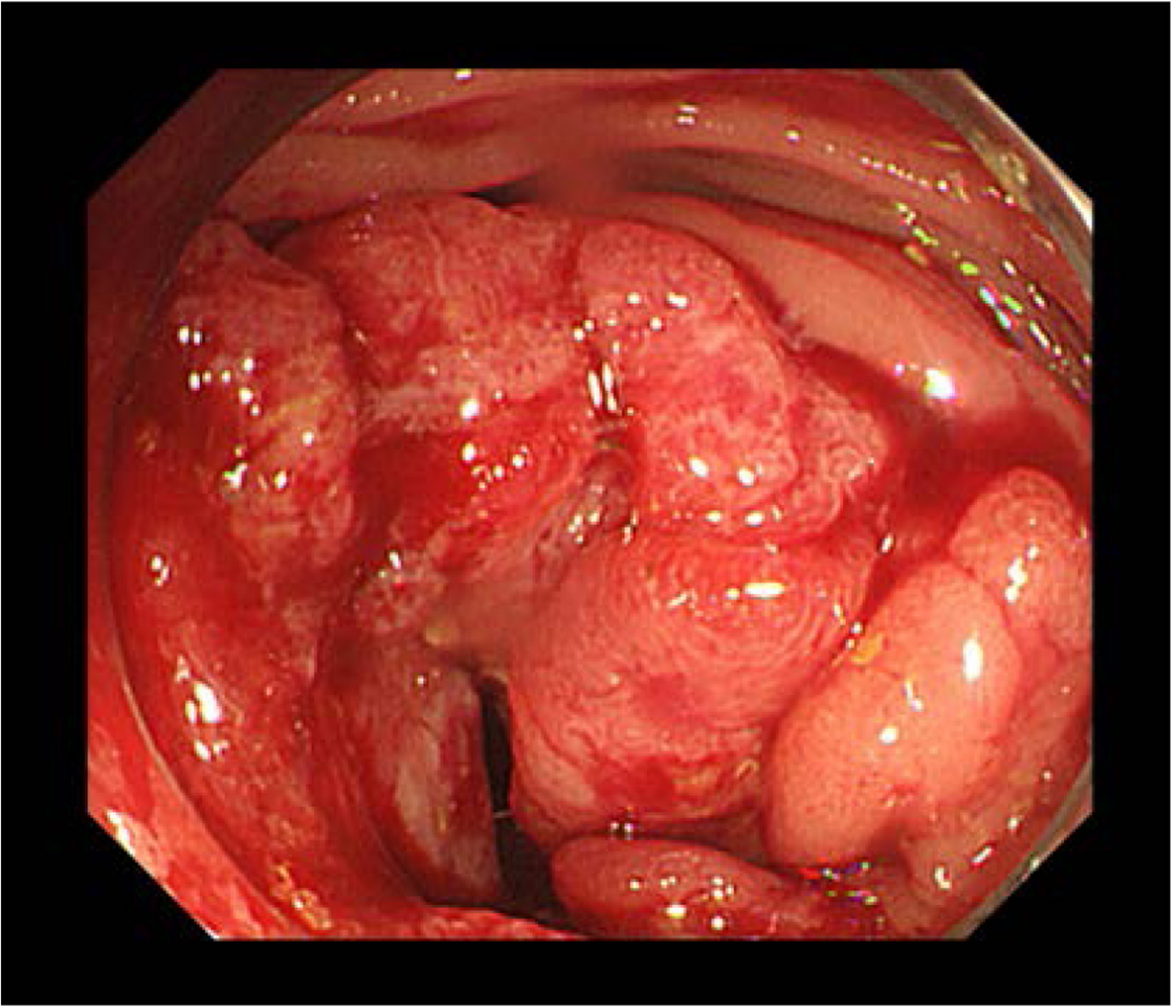
Colonoscopy showed a circumferential tumor in the transverse colon

**Fig. 2:**
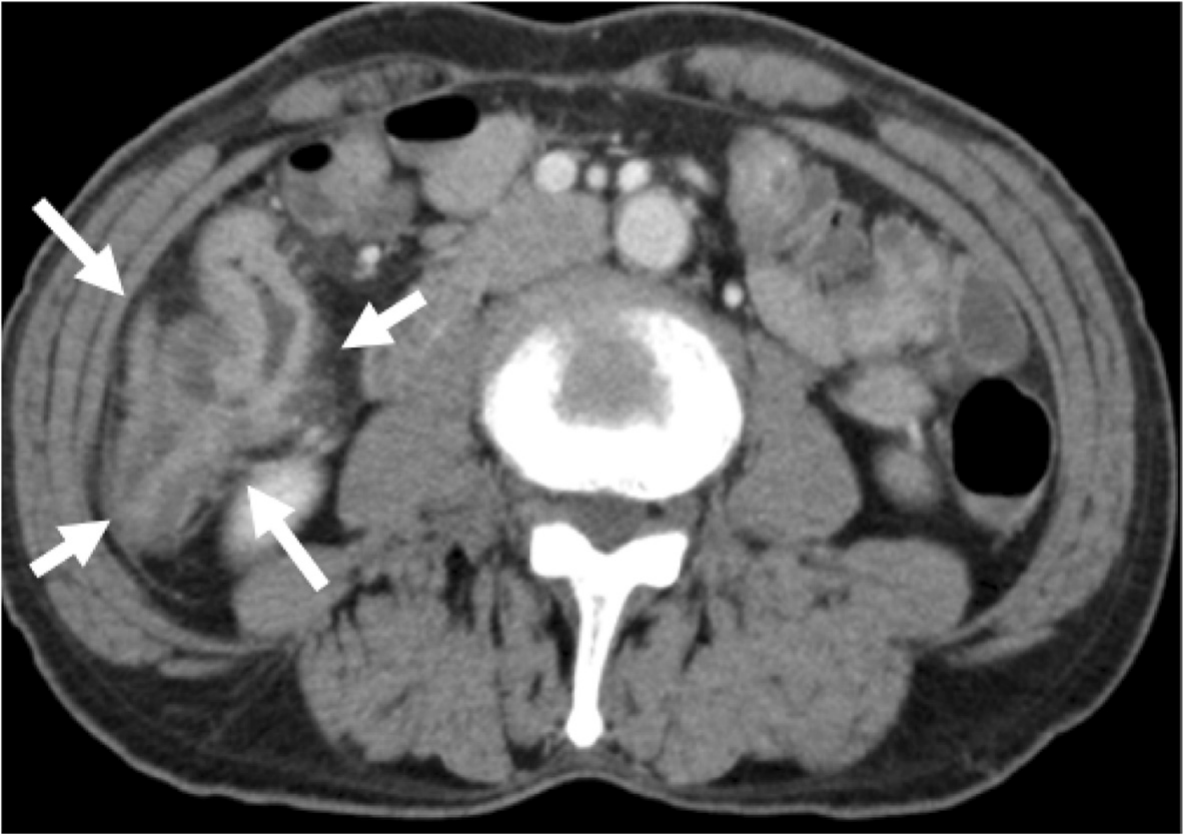
Abdominal enhanced computed tomography showed a sigmoid colostomy (because of Miles operation), a tumor located in the hepatic flexure (*white arrows* show), and lymph node metastasis. However, distant metastasis was not observed

**Fig. 3:**
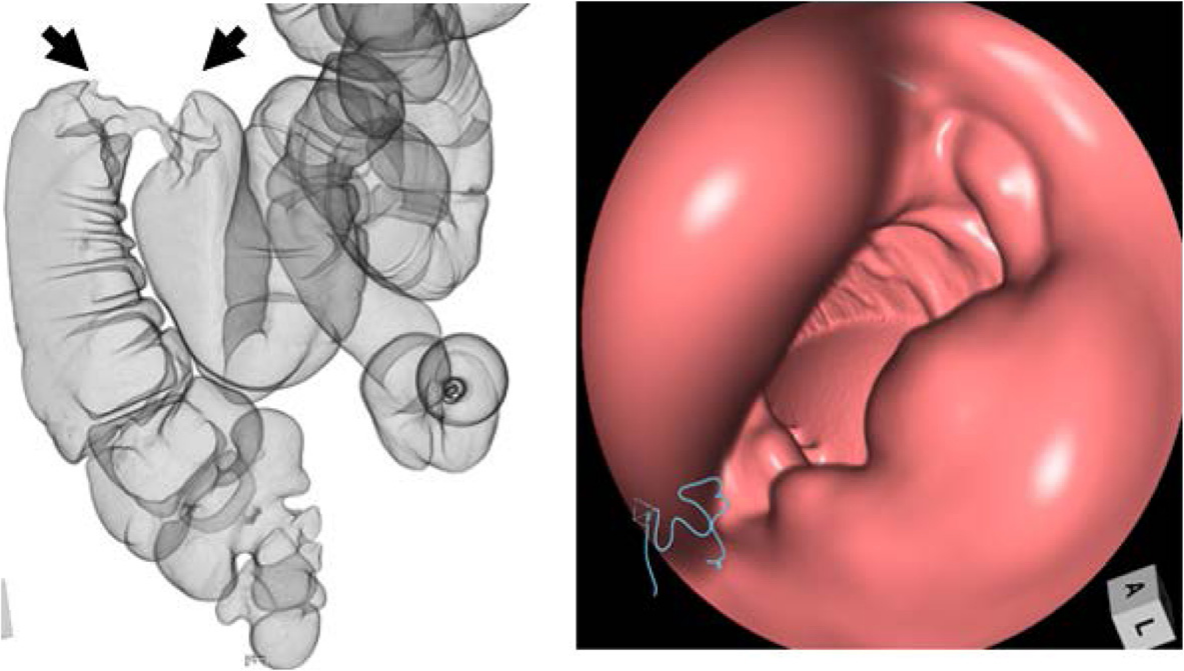
Computed tomographic colonography revealed the apple core sign in the hepatic flexure (*black arrows* show), without any evident tumor in the proximal colon

A 68-year-old man was admitted to our hospital with right abdominal pain and bloody stool that had been present for 2 weeks prior to admission. His medical history included abdominoperineal rectal resection with permanent sigmoid stoma (Miles operation), which had been performed 25 years earlier. On admission, assessments of tumor markers revealed a carcinoembryonic antigen (CEA) level of 1.2 ng/mL (normal limits 0.1–5.0 ng/mL) and a carbohydrate antigen 19-9 (CA19-9) level of 3.0 U/mL (normal limits 0.2–37.0 U/mL). Colonoscopy showed a circumferential tumor in the transverse colon but provided no information about the proximal colon because of the presence of this occlusive tumor (Fig. 1). Histopathological analysis of the biopsy specimen revealed tubular adenocarcinoma. Abdominal enhanced computed tomography revealed a tumor located in the hepatic flexure and local lymph node metastasis. However, distant metastasis was not observed (Fig. 2). Accordingly, we considered the cancer to be resectable, although the investigation of the proximal colon was not sufficient to proceed. Thus, CTC was planned to assist our examination of the proximal colon under sigmoid colostomy. During CTC, the catheter was carefully inserted until the balloon portion was located several centimeters past the stoma. A 25-mL balloon inflation volume was employed. During the examination, the pressure of the colon was monitored through the catheter to prevent perforation and luminal collapse. Because of the patient’s stoma, he had difficulty lying in the prone position. Therefore, he lay in a standard supine position and a right-sideways position during CT scanning (Aquilion ONE 320, Toshiba, Tokyo). CTC revealed the apple core sign in the hepatic flexure, without any evident tumor in the proximal colon (Fig. 3). Therefore, we performed transverse colectomy and lymph node dissection, preserving a part of the ascending colon and Bauhin valve. Histopathology revealed a tubular adenocarcinoma of the transverse colon (R0, T4N0P0H0M0, stage II). Postoperative colonoscopy confirmed that the ascending colon did not contain any lesion. The patient has remained free of recurrence for 2 years.

### Discussion

Here, we have described a case in which CTC was used to evaluate a transverse carcinoma after Miles operation. Based on our experience with this case, we would like to emphasize two points.

First, CTC allowed the proximal colon to be investigated effectively, even though the colonoscopy was incomplete because of a circumferential tumor. Preoperative evaluation of the entire colon is important in patients with colorectal cancer because the identification of synchronous cancers, which are present in 1–7 % of these patients, may determine the extent of surgical resection [6, 7]. Several methods of examination are useful for proximal colonic evaluation, including double-contrast barium enema, CTC, and surgical palpation. However, double-contrast barium enema has low sensitivity [8]. Moreover, patients receiving barium enema are exposed to much more radiation than are patients undergoing CTC [9]. On the other hand, CTC is known to be a safe procedure, particularly when it is performed using low-pressure carbon dioxide insufflation; reported rates of overall procedure-related colonic perforations range from 0.009 to 0.06 % [10]. So, CTC is currently regarded as the standard procedure in patients with sub-occlusive tumors.

However, the clinical practice of CTC remains somewhat unclear in patients with sub-occlusive colorectal cancer. CTC usually requires high-dose bowel preparations in order to distinguish polyps from stool, yet these high-dose bowel preparations are difficult for patients with sub-occlusive cancer. To be safe, patients at our hospital with sub-occlusive colorectal cancer only take highly fluid diet such as enteral nutrition (from 3 to 7 days) before CTC. Patients subsequently undergo CTC using low-dose bowel preparations.

Second, we would also like to emphasize that CTC was conducted in a technically safe manner, although our patient had a sigmoid colostomy. Lee et al. have reported the feasibility of CTC in 18 patients with colostomy. Examinations were performed uneventfully for all but one patient, who developed temporary air and fluid leakage. Four patients (22.2 %) had segments that were not adequately visualized in either position, owing to luminal collapse; all of these segments were in the sigmoid colon. Three patients (16.7 %) had areas that were submerged under fecal matter in both positions, but these areas were evaluable because of fecal tagging or IV contrast enhancement [11].

**Fig. 4:**
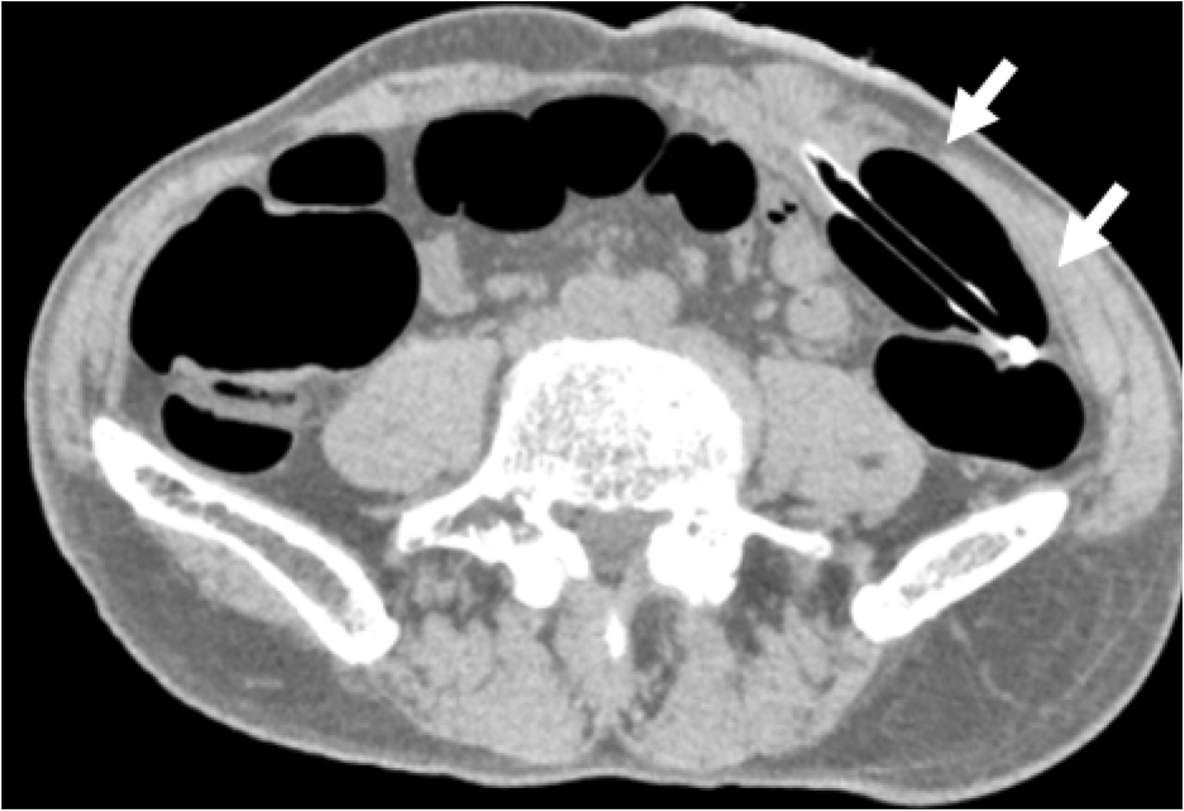
The catheter was placed until the balloon portion was located several centimeters past the stoma (*white arrows* show)

Despite these feasibility results, CTC examinations of patients with colostomy require several unique techniques. During the examination, patients with colostomy more frequently develop air leakage and expulsion of the balloon catheter because the rectal sphincter is absent in artificial colostomy. Therefore, the catheter was carefully inserted until the balloon portion was located several centimeters past the stoma. This balloon location was chosen to avoid ballooning in the stoma and to ensure some distance between entrance of the stoma and ballooning position (Fig. 4). A 25-mL balloon inflation volume was employed. For these procedures, patients lay in a standard supine position and a right-sideways position during CTC examination, because patients with colostomy have difficulty in prone positions due to the pouch of the stoma. In our case, during the examination, we monitored the pressure of the intestine to prevent perforation and the expulsion of the catheter.

## Conclusions

CTC examination can be an effective means of preoperatively evaluating the proximal colon in patients with occlusive tumor. Further, CTC examination was technically feasible through a sigmoid stoma.

### Consent

Written informed consent was obtained from the patient for publication of this case report and any accompanying images. A copy of the written consent is available for review by the Editor-in-Chief of this journal.

## Abbreviations

CT: computed tomography
CTC: computed tomographic colonography
